# Urinary Bisphenol A and Type-2 Diabetes in U.S. Adults: Data from NHANES 2003-2008

**DOI:** 10.1371/journal.pone.0026868

**Published:** 2011-10-26

**Authors:** Monica K. Silver, Marie S. O'Neill, MaryFran R. Sowers, Sung Kyun Park

**Affiliations:** 1 Department of Environmental Health Sciences, School of Public Health, University of Michigan, Ann Arbor, Michigan, United States of America; 2 Department of Epidemiology, School of Public Health, University of Michigan, Ann Arbor, Michigan, United States of America; Brigham & Women's Hospital and Harvard Medical School, United States of America

## Abstract

**Objective:**

Bisphenol A (BPA) is found in plastics and other consumer products; exposure may lead to insulin resistance and development of type-2 diabetes mellitus (T2DM) through over-activation of pancreatic β-cells. Previous studies using data from the National Health and Nutrition Examination Survey (NHANES) showed an inconsistent association between prevalence of self-reported T2DM and urinary BPA. We used a different diagnosis method of T2DM (hemoglobin A1c (HbA1c)) with a larger subset of NHANES.

**Methods and Findings:**

We analyzed data from 4,389 adult participants who were part of a sub-study of environmental phenol measurements in urine from three NHANES cycles from 2003 to 2008. T2DM was defined as having a HbA1c ≥6.5% or use of diabetes medication. The weighted prevalence of T2DM was 9.2%. Analysis of the total sample revealed that a two-fold increase in urinary BPA was associated with an odds ratio (OR) of 1.08 of T2DM (95% confidence interval (CI), 1.02 to 1.16), after controlling for potential confounders. However, when we examined each NHANES cycle individually, we only found a statistically significant association in the 2003/04 cycle (n = 1,364, OR = 1.23 (95% CI, 1.07 to 1.42) for each doubling in urinary BPA). We found no association in either the NHANES cycle from 2005/06 (n = 1,363, OR = 1.05 (95% CI, 0.94 to 1.18)); or 2007/08 (n = 1,662, OR = 1.06 (95% CI, 0.91 to 1.23)). Similar patterns of associations between BPA and continuous HbA1c were also observed.

**Conclusions:**

Although higher urinary BPA was associated with elevated HbA1c and T2DM in the pooled analysis, it was driven by data from only one NHANES cycle. Additional studies, especially of a longitudinal design with repeated BPA measurements, are needed to further elucidate the association between BPA and T2DM.

## Introduction

Bisphenol A (BPA) is a high-volume production chemical used worldwide in the manufacturing of polycarbonate plastics including numerous consumer products like food and water containers and bottles. BPA is also found in the resin linings of food and beverage cans and dental sealants [1], leaching readily from many of these products and leading to exposure in large segments of the population [2]. Biomonitoring data indicate that 93% of U.S. general population aged six and older has detectable levels of BPA in urine [3].

While BPA has been evaluated as an endocrine disruptor, the potential metabolic effects of BPA are also of interest. Studies using rodent models have suggested that BPA can alter insulin biosynthesis and secretion in pancreatic β-cells, potentially through the over-activation of the estrogen receptor, ERα [4]–[6]. This may lead to insulin resistance and the subsequent development of type-2 diabetes mellitus (T2DM). Other evidence of BPA's metabolic effects include dysregulation of glucose transport in adipocytes [7] and inhibition of adiponectin release [8].

Previous epidemiological studies using data from the National Health and Nutrition Examination Survey (NHANES), which combines questionnaires and physical exams to assess health and nutrition in the U.S. population (http://www.cdc.gov/nchs/nhanes.htm) have shown an inconsistent association between the prevalence of self-reported T2DM and urinary BPA levels. In NHANES 2003–2004, T2DM was positively associated (odds ratio (OR) = 1.39; 95% confidence interval (CI), 1.21 to 1.60) with a 1 standard deviation increase in BPA) [9], but this association was not found in the subsequent cycle of NHANES 2005–2006 (OR = 1.02; 95% CI, 0.76 to 1.38) [10].

Although self-reported diabetes is reported to be reasonably in agreement with medication use and the clinical cutoff, as determined by fasting glucose levels (126 mg/dL or higher) [11]–[13], the possibility of an underestimation of diabetes in the population by using this outcome measure still exists, since people may not be aware of their true clinical status [14]. Recently, the International Expert Committee recommended the use of the hemoglobin A1c (HbA1c), a measure of glycated hemoglobin in red blood cells, as an alternative method for the diagnosis of diabetes [15].

In addition to potential problems with the diabetes outcome metric used in previous studies, those previous studies also assumed linear exposure-response relations between urinary BPA and the health outcomes including T2DM [9], [10]. Non-linear exposure-response relations are frequently observed in environmental epidemiologic studies when biomonitoring exposure data are used. Often, a higher slope in the lower exposure region and a plateau in the higher exposure region (log-linear exposure-response) is observed [16]. Therefore, a more thorough exploration of the shape of the exposure-response curve may be warranted to aid assessment of the risks BPA may pose to human health, and in particular, metabolic function.

Given the increasing burden of T2DM and the ubiquitous exposures to BPA, determining if the two are associated may have important implications for prevention. Here, we examined the possible association between BPA exposure and T2DM, defined as HbA1c greater than and equal to 6.5%, in an expanded NHANES population combining three independent cycles from 2003 to 2008 as well as in individual cycles. We also explored exposure-response relations using a smoothing method (natural splines).

## Methods

### Ethics Statement

NHANES is a publicly available data set and all participants in NHANES provide written informed consent, consistent with approval by the National Center for Health Statistics Institutional Review Board.

### Study Population

Data were from the U.S. NHANES 2003/04, 2005/06, and 2007/08 study cycles (continuous NHANES) [17]. NHANES is a cross-sectional study designed to be representative of the health and diet of the non-institutionalized U.S. population. NHANES employs a stratified sampling design with accompanying design weights. For the present study, 4,792 adults, aged 20 years or older, who participated in the sub-study of the environmental phenol measurement in urine, were eligible. Eight subjects with urinary BPA concentrations greater than 80.1 ng/mL were excluded, consistent with previous reports [9], [10]. An additional 403 subjects were excluded because data were missing for important covariates, decreasing the final sample size to 4,389 subjects (1,364 from NHANES 2003/04; 1,363 from NHANES 2005/06; 1,662 from NHANES 2007/08).

### Urinary BPA

A one-third random subset of NHANES subjects was selected and asked to provide spot urine samples for subsequent laboratory analysis. Total urinary BPA, both conjugated and free, was measured using online solid-phase extraction coupled to high performance liquid chromatography (HPLC)-isotope dilution tandem mass spectrometry at the Division of Environmental Health Laboratory Sciences (National Center for Environmental Health, CDC). Extensive quality assurance and quality control (QC) procedures were followed, including confirmation that samples were not contaminated during collection, handling and analysis [18], [19]. The coefficients of variation were 8.1–18.6% for the low BPA QC pools and 5.7–12.1% for the high BPA QC pools. The lower limit of detection (LLOD) was 0.36 ng/mL in 2003/04 and 0.4 ng/mL in 2005/06 and 2007/08 NHANES cycles. Urinary BPA concentrations below LLOD were found in 8.5%, 9.6% and 7.8% of the participants in the 2003/04, 2005/06 and 2007/08 cycles, respectively. For these participants, we substituted a value equal to the LLOD divided by the square-root of two. To be consistent with the previous study by Melzer et al. [10], we assigned the value of LLOD/√2 (0.28 ng/mL) used in the 2005/06 and 2007/08 cycles to those participants from the NHANES 2003/04 cycle.

### Hemoglobin A1c and T2DM

HbA1c was measured with HPLC by the Diabetes Diagnostic Laboratory at the University of Missouri-Columbia using Primus CLC 330 and Primus CLC 385 (Primus Corporation, Kansas City, MO) in the 2003/04 cycle and the Diabetes Laboratory at the University of Minnesota using Tosoh A1c 2.2 PlusGlycohemoglobin Analyzer (Tosoh Medics, Inc., San Francisco, CA) in the 2005/06 and 2007/08 cycles. Two types of QC were used: 1) batch QC specimens placed in each run, and 2) sample QC specimens (2% of the total specimens) were randomly selected from each run and analyzed in a second run. The coefficients of variation were 1.4–1.7% for normal HbA1c QC pools and 0.8–1.1% for elevated HbA1c QC pools in all three NHANES cycles. A crossover calibration study to compare the 2005/06 Tosoh method to the 2003/04 Primus method revealed a significant difference [20] which required the application of the following adjustment, Y (Tosoh) = -0.5279 + 1.0781*X (Primus), prior to undertaking statistical analyses. T2DM was defined as HbA1c ≥6.5% or self-reported use of diabetes medication (insulin or blood sugar-lowering pills).

### Other covariates

Demographic information, medical history and physical and laboratory assessment were obtained by self-report, computer-assisted personal interviewing and physical examination, respectively. Gender was categorized as male or female. Race/ethnicity was categorized into five groups: Mexican American, other Hispanic, non-Hispanic White, non-Hispanic Black, and other. Self-reported educational attainment was categorized into three levels: <high school, high school diploma, and some college and over. Self-reported annual household income was categorized into five levels: <$20,000, $20,000 to $34,999, $35,000 to $64,999, $65,000 and over, and unknown income (“over $20,000” (n = 92, 2% of the sample) was classified into unknown). Smoking status was categorized into never, former, and current occasional and daily smokers. Body mass index (BMI) was calculated as measured weight in kilograms divided by measured height in meters squared. Waist circumference was measured in centimeters. Urinary creatinine, an indicator of renal status, was measured on the Beckman CX3 using a Jaffe reaction in the NHANES 2003/04 and 2005/06 cycles and on the Roche ModP using an enzymatic (creatinase) method in the NHANES 2007/08 cycle. Because the Jaffe method is subject to more interference, NHANES conducted a crossover study to identify the following equations to adjust urinary creatinine assayed before 2007 [21]:

Urine creatinin e<75 mg/dL:
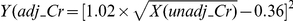



Urine creatinine 75 to<250 mg/dL:
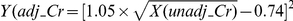



Urine creatinine ≥250 mg/dL:
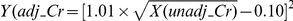



These adjustments were applied for the current analysis.

### Statistical Analysis

NHANES implemented a complex cluster sample design to include adequate representation from various socioeconomic strata and minorities. In recognition of these design effects, we computed weighted estimates according to the NHANES Analytic and Reporting Guidelines [22] including six-year sampling weights. We used the survey package (version 3.22-3) in R software (version 2.11.1; R Foundation for Statistical Computing, http://www.r-project.org).

The distribution of urinary BPA was highly right-skewed, and thus, we computed survey-weighted geometric means and geometric standard errors (SEs). We used survey-weighted generalized linear models to fit binary T2DM and continuous HbA1c outcomes. The following variables were chosen *a priori* as covariates in the models because of their biological relevance to the outcomes: age (linear and quadratic terms), gender, race/ethnicity, education, income, BMI, waist circumference, smoking status and urinary creatinine [3], [9], [10], [23]. We modeled urinary creatinine with a natural spline (restricted cubic spline) with four degrees of freedom (knots at 25^th^, 50^th^, and 75^th^ percentiles) to accommodate potential nonlinearity because log-transformation or adding a quadratic term could not capture such nonlinearity well. We constructed sequential covariate models: model 1 adjusted for urinary creatinine and age; model 2: further adjustment for gender, race-ethnicity, education and income; model 3 further adjustment for smoking status, BMI, and waist circumference. BMI and waist circumference appeared collinear but we kept both in the model because the effect estimate of urinary BPA was not influenced by fitting either variable and in order to make our model comparable to the models of the previous two NHANES-based studies [9], [10].

**Figure 1:**
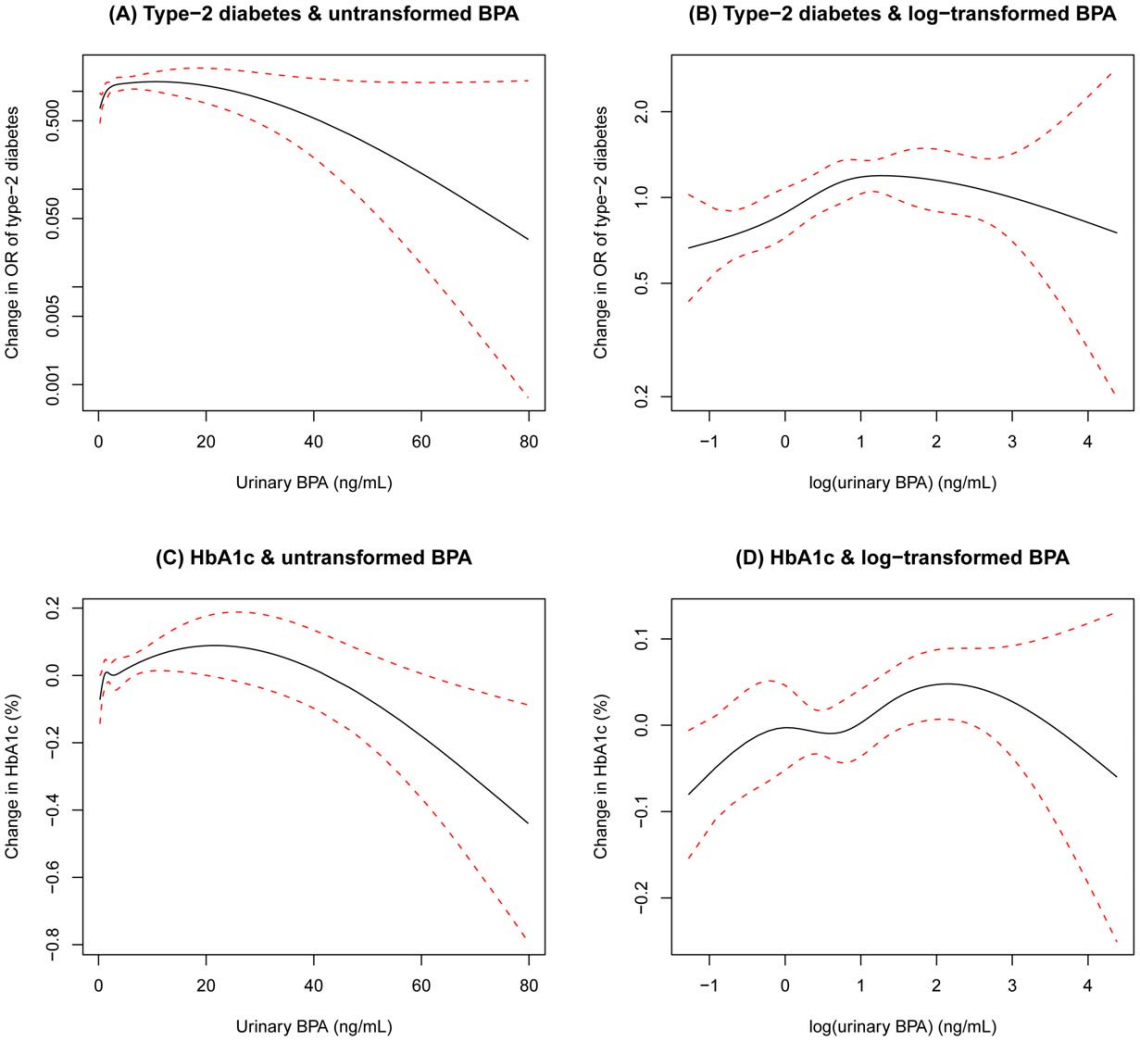
Associations of type-2 diabetes and hemoglobin A1c (HbA1c) with untransformed and log-transformed urinary bisphenol A (BPA), adjusted for urinary creatinine (natural spline (restricted cubic spline) with four degrees of freedom (knots at 25^th^, 50^th^, and 75^th^ percentiles)), age, age^2^, gender, race-ethnicity, education, household income, body mass index, waist circumference, and smoking status. The solid line indicates the smoothing trends estimated from the natural spline (restricted cubic spline) with four degrees of freedom (knots at 25^th^, 50^th^, and 75^th^ percentiles), and the dotted lines indicate its 95% confidence intervals.

We examined the shapes of dose-response relations by fitting the term for BPA using natural splines with four degrees of freedom. Graphs of these natural splines showed that the association between urinary BPA and both T2DM and HbA1c was close to a log-linear association (Figure 1), so we chose a log-transformation of urinary BPA for the final statistical model.

To evaluate the consistency of the relationships across the NHANES cycles, we repeated the same regression models for each cycle individually, using each cycle's sampling weight. Finally, we assessed for effect modification by age (20–39/40–59/≥60 years), gender, race/ethnicity, education, household income, smoking status and BMI (<25/25–29.9/≥30 kg/m^2^) by adding an interaction term between log-transformed urinary BPA and the corresponding characteristics, along with the main effects from model 3. We obtained p-values for linear trend of ordinal variables (age, education, household income and BMI), as well as p-values for comparisons of the other categorical variables.

## Results

Table 1 shows sample-weighted characteristics of study participants in the pooled data and by individual NHANES cycle. The geometric means of urinary BPA and creatinine-corrected BPA were 2.0 (95% CI, 1.9 to 2.1) ng/mL and 2.1 (95% CI, 2.0 to 2.2) ng/mg, respectively. The mean level of HbA1c was 5.5% (SE = 0.02). The prevalence of T2DM was 9.2%. The mean age was 46.5 years (SE = 0.36) and 51.5% of the sample were females. There was a statistically significant difference in the urinary BPA concentrations across cycles. The geometric means of urinary BPA were 2.4 ng/mL (95% CI, 2.1 to 2.7) in NHANES 2003/04, 1.7 ng/mL (95% CI, 1.6 to 1.9) in NHANES 2005/06, and 2.0 ng/mL (95% CI, 1.8 to 2.1) in NHANES 2007/08 (p<0.0001). The HbA1c level in NHANES 2007/08 (5.6%) was significantly higher than that of the other two cycles (5.4%) (p<0.001), and there was a marginally significant difference in the prevalence of T2DM by the cycle (7.8%, 9.0%, 10.7% from NHANES 2003/04 to 2007/08, respectively; p = 0.06). The un-calibrated HbA1c level in NHANES 2003/04 was 5.5% (data not shown). Urinary BPA concentrations were higher in younger adults, males, participants with lower income, and current smokers (Table 2).

**Table 1:** Characteristics of participants (weighted mean ± standard error or weighted percentage) by NHANES cycle, National Health and Nutrition Examination Survey, 2003-2008.

		NHANES cycle			
	Pooled	2003/04	2005/06	2007/08	P*
N	4389	1364	1363	1662	
Age (years)	46.5±0.36	46.1±0.48	46.6±0.87	46.9±0.46	0.64
Female (%)	51.5	50.1	51.9	52.4	0.60
Race/ethnicity (%)					
Non-Hispanic White	72.0	72.4	72.9	70.8	0.91
Non-Hispanic Black	10.4	9.8	11.3	10.3	
Mexican American	7.9	7.3	7.6	8.7	
Other Hispanic	4.0	4.1	3.2	4.8	
Other	5.6	6.4	5.0	5.5	
Education (%)					
Less than high school	17.9	17.7	16.3	19.6	0.53
High school diploma	25.3	25.1	24.8	26.1	
Some college+	56.8	57.2	58.9	54.3	
Household income (%)					
<$20,000	14.2	15.6	13.3	13.7	0.31
$20,000 to $34,999	17.7	18.2	17.7	17.3	
$35,000 to $64,999	25.2	27.2	25.7	22.7	
≥$65,000	37.7	33.4	38.8	40.6	
Unknown	5.3	5.5	4.5	5.7	
Cigarette smoking (%)					
Never	51.1	50.6	50.1	52.7	0.45
Former	24.9	25.2	24.2	25.3	
Current	24.0	24.2	25.6	22.1	
Body mass index (kg/m^2^)	28.4±0.13	28.0±0.23	28.5±0.24	28.8±0.21	0.07
Waist circumference (cm)	97.8±0.37	97.1±0.50	97.6±0.75	98.7±0.62	0.17
Urinary creatinine (mg/dL)†	96.4 (93.0, 100)	97.4 (90.7, 105)	95.5 (88.9, 103)	96.4 (91.6, 101)	0.92
Urinary bisphenol A (ng/mL)†	2.0 (1.9, 2.1)	2.4 (2.1, 2.7)	1.7 (1.6, 1.9)	2.0 (1.8, 2.1)	<0.001
Cr-corrected BPA (ng/mg)†	2.1 (2.0, 2.2)	2.4 (2.2, 2.7)	1.8 (1.7, 1.9)	2.0 (1.9, 2.2)	<0.001
Hemoglobin A1c (%)	5.5±0.02	5.4±0.03	5.4±0.04	5.6±0.03	<0.001
Diabetes medication (%)	7.0	6.0	6.9	8.1	0.14
Diabetes (%)‡	9.2	7.8	9.0	10.7	0.06

*p-value based on the Rao-Scott log-likelihood ratio test for continuous variables and the Rao-Scott Chi-square test for categorical variables.

† Geometric mean (95% confidence interval) is presented.

‡ Defined as HbA1c ≥6.5% or use of diabetes medication.

**Table 2:** Geometric mean of urinary bisphenol A concentrations by participant characteristics.

	N	Weighted percentage	Weighted GM (95% CI)	p-value*
All	4389	100	2.0 (1.9, 2.1)	
Age (years)				
20–39	1537	37.4	2.4 (2.3, 2.6)	0.002
40–59	1389	40.0	1.9 (1.7, 2.0)	
60+	1463	22.6	1.6 (1.5, 1.8)	
Gender				
Males	2117	48.5	2.2 (2.1, 2.3)	<.0001
Females	2272	51.5	1.8 (1.7, 2.0)	
Race/ethnicity				
Non-Hispanic White	2227	72.0	1.9 (1.8, 2.0)	
Non-Hispanic Black	865	10.4	2.9 (2.7, 3.2)	0.40
Mexican American	846	7.9	2.1 (1.9, 2.3)	0.41
Other Hispanic	270	4.0	2.3 (2.0, 2.7)	0.19
Other	181	5.6	1.6 (1.3, 1.9)	0.10
Education				
Less than high school	1226	17.9	2.1 (1.9, 2.3)	0.85
High school diploma	1087	25.3	2.1 (1.9, 2.3)	
Some college+	2076	56.8	2.0 (1.8, 2.1)	
Household income				
<$20,000	940	14.2	2.3 (2.1, 2.6)	0.0005
$20,000 to $34,999	920	17.7	2.1 (1.9, 2.3)	
$35,000 to $64,999	1071	25.2	2.1 (1.8, 2.3)	
≥$65,000	1192	37.7	1.8 (1.7, 2.0)	
Unknown	266	5.3	2.1 (1.8, 2.5)	
Smoking status				
Never	2295	51.1	2.0 (1.9, 2.1)	
Former	1116	24.9	1.9 (1.7, 2.1)	0.37
Current	978	24.0	2.2 (2.0, 2.4)	0.004
Body mass index (kg/m^2^)				
<25	1339	33.8	1.8 (1.6, 1.9)	0.80
25-29.9	1475	31.9	2.0 (1.8, 2.1)	
30+	1575	34.3	2.3 (2.1, 2.5)	
Diabetes				
No	3849	90.8	2.0 (1.9, 2.1)	0.32
Yes	540	9.2	2.1 (1.9, 2.3)	

*p-value based on the test for linear trend for ordinal variables (age, education, household income (excluding Unknown), and body mass index) and the test for difference compared with the reference group for the other categorical variables (cycle, gender, race/ethnicity, smoking status, and diabetes).

Table 3 shows the logistic regression results for models with various sets of covariate in the pooled data and by each cycle. After adjusting for urinary creatinine, age, gender, race-ethnicity, education, household income, BMI, waist circumference, and smoking status (model 3), the ORs for T2DM for a two-fold increase in urinary BPA were 1.08 (95% CI, 1.02 to 1.16), 1.23 (95% CI, 1.07 to 1.42), 1.05 (95% CI, 0.94 to 1.18), and 1.06 (95% CI, 0.91 to 1.23) in the pooled data, NHANES 2003/04, 2005/06, and 2007/08 cycles, respectively. The associations between urinary BPA and T2DM seemed to be robust to the influences of the demographic variables, obesity and smoking (Table 3, models 1 and 2). Similar associations were found when urinary BPA and continuous HbA1c outcome was examined (Table 4). There were statistically significant associations in the pooled data (0.017% change (95% CI, 0.001% to 0.032%) in HbA1c for a doubling in urinary BPA in model 3), and in the NHANES 2003/04 cycle (0.048% change in HbA1c (95% CI, 0.019% to 0.076%)), but we did not observe statistically significant associations for either the NHANES 2005/06 or 2007/08 cycles.

**Table 3:** Odds Ratios (95% confidence intervals) of type-2 diabetes for a doubling in urinary bisphenol A concentrations.

		NHANES cycle		
	Pooled	2003/04	2005/06	2007/08
Crude	1.02 (0.97, 1.08)	1.06 (0.94, 1.20)	1.06 (0.95, 1.18)	0.98 (0.90, 1.07)
Model 1	1.08 (1.01, 1.14)	1.20 (1.06, 1.35)	1.07 (0.98, 1.17)	1.03 (0.92, 1.17)
Model 2	1.09 (1.02, 1.16)	1.23 (1.07, 1.42)	1.08 (0.98, 1.19)	1.04 (0.90, 1.21)
Model 3	1.08 (1.02, 1.16)	1.23 (1.07, 1.41)	1.06 (0.95, 1.19)	1.06 (0.91, 1.23)

Model 1: age, age^2^, urinary creatinine as natural splines (restricted cubic splines) with 4 degrees of freedom (knots at 25^th^, 50^th^, and 75^th^ percentiles).

Model 2: Further adjusted for gender, race-ethnicity, education, and household income.

Model 3: Further adjusted for body mass index, waist circumference, and smoking status.

**Table 4:** Linear regression coefficients (95% confidence intervals) of hemoglobin A1c (%) for a doubling in urinary bisphenol A concentrations.

		NHANES cycle		
	Pooled	2003/04	2005/06	2007/08
Crude	-0.003 (-0.017, 0.012)	0.015 (-0.006, 0.036)	0.010 (-0.019, 0.039)	-0.026 (-0.054, 0.001)
Model 1	0.020 (0.005, 0.034)	0.049 (0.027, 0.072)	0.020 (-0.007, 0.046)	0.001 (-0.022, 0.024)
Model 2	0.022 (0.008, 0.036)	0.055 (0.032, 0.078)	0.020 (-0.006, 0.046)	0.002 (-0.022, 0.027)
Model 3	0.017 (0.001, 0.032)	0.048 (0.019, 0.076)	0.014 (-0.015, 0.042)	-0.001 (-0.023, 0.022)

Model 1: age, age^2^, urinary creatinine as natural splines (restricted cubic splines) with 4 degrees of freedom (knots at 25^th^, 50^th^, and 75^th^ percentiles).

Model 2: Further adjusted for gender, race-ethnicity, education, and household income.

Model 3: Further adjusted for body mass index, waist circumference, and smoking status.

**Figure 2:**
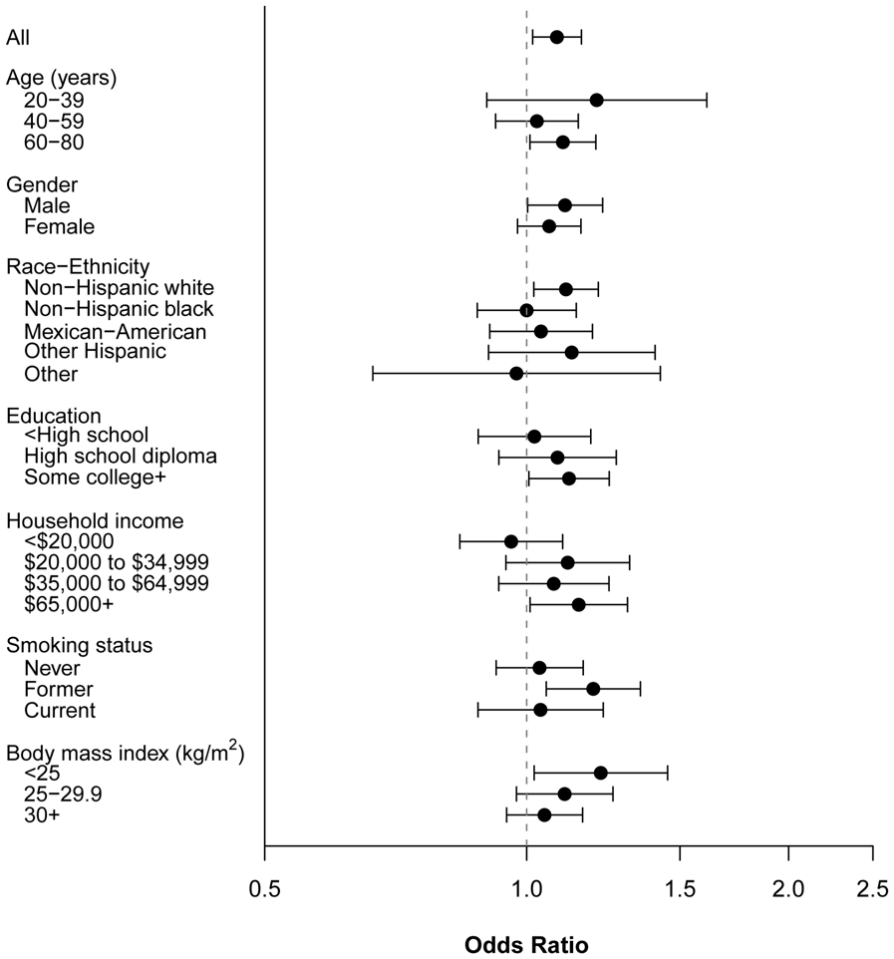
Adjusted odds ratio of type-2 diabetes for a doubling in urinary bisphenol-A (BPA) by important subgroups. The adjusted covariates are the same as in Figure 1.

Figure 2 shows results of the effect modification analysis in the association between urinary BPA and T2DM by participant characteristics. No effect modification by age, gender, race-ethnicity, education, household income, smoking status, or BMI was observed.

## Discussion

With remarkable increases in prevalence, T2DM is considered an emerging pandemic in the 21^st^ century and is an important public health concern. Recently, attention has been paid to the potential contribution of BPA to the etiology of this disease, and the study by Lang et al., that reported statistically significant associations between urinary BPA and self-reported, physician-diagnosed heart disease and T2DM in the NHANES 2003/04 cycle [9], has catalyzed a hot debate on the potential role of BPA as a “diabetogen” [24]. The present work complements the analyses of Lang et al. [9] and Melzer et al. [10] by further examining the question of whether or not BPA exposure may have an adverse impact on glucose homeostasis, and by further expanding the NHANES cycles through 2007/08. HbA1c was used as a biomarker for glucose homeostasis and T2DM, rather than the previously studied, self-reported, physician-diagnosed diabetes measurement.

Here, we found that urinary BPA concentrations were significantly positively associated with the prevalence of T2DM as well as the continuous measure of HbA1c when all three NHANES cycles were combined and analyzed together. However, when we completed the analyses separately for each NHANES cycle, we only found a statistically significant association between urinary BPA and T2DM and HbA1c in the NHANES 2003/04 cycle. Further, there was no consistency in the association among different cycles. This suggests that the observed, statistically significant association in the pooled data may actually be driven by data from only one NHANES cycle. This may, in part, be due to the significantly higher mean levels of BPA found in 2003/04 (2.4 ng/mL) versus 2005/06 (1.7 ng/mL) and 2007/08 (2.0 ng/mL). It is unclear whether this difference is due to random chance or some variation in the sampling of the different cycles. It is unlikely that the significantly lower mean urinary BPA concentrations for 2005–2008 are due to a decrease in exposure to BPA, since it is only recently that BPA has captured the public's attention thus leading to an increased number of BPA-free products on the market. It may be most likely, however, that the non-significant associations in 2005/06 and 2007/08 cycles were due to lowered statistical power as a result of the narrower range (less variation) of urinary BPA (95% CI, 2.1 to 2.7 ng/mL in 2003/04 versus 1.6 to 1.9 ng/mL in 2005/06 and 1.8 to 2.1 ng/mL in 2007/08, Table 1). Graphical examination using natural splines revealed that the exposure-response relationships were close to being log-linear rather than linear (Figure 1). This suggests that the assumption of linearity in the association between BPA and T2DM made in previously published work may not be appropriate.

We chose HbA1c as the outcome for this study for two reasons. First, HbA1c is relatively more stable compared to other markers of glycemic indices, such as fasting blood glucose, because it provides a measure of average blood glucose over the previous two to three months [25]. Second, a continuous biomarker such as HbA1c would better capture individuals with undiagnosed disease, which could reduce outcome misclassification. Seven million people in the U.S. are believed to have undiagnosed diabetes [26]. The absence of diagnosis is likely the biggest concern in underserved populations, those of lower socioeconomic status, and those with poorer access to healthcare. A study of newly-admitted hospital patients in the Bronx, New York found that 35% of patients had HbA1c levels that met the clinical definition of T2DM but had never been diagnosed [27]. Therefore, we considered HbA1c a favorable outcome measure for this study because it would include individuals without a physician's diagnosis who might otherwise have been excluded.

Some diabetes researchers and physicians argue that several factors could potentially lead to inaccuracies in the HbA1c test and a subsequent misdiagnosis. These include any kind of hemoglobinopathy, such as sickle cell anemia or other anemias, which tend to be found more frequently in subjects of selected race/ethnicity, gender or age [25], [28]. This possibility is unlikely to have influenced our results, because age, gender and race-ethnicity did not modify the association between urinary BPA and T2DM in the present study. An additional issue with HbA1c is that there seem to be inconsistencies across laboratory equipment and methods so that results vary based on the laboratory performing the test [25], [28]. In NHANES, the laboratory methods for HbA1c were changed between 2003/04 (Primus method) and 2005/06 (Tosoh method). One may argue that the statistically significant association found only in the NHANES 2003/04, but not in the other two cycles, is due to the difference in the assay method for HbA1c. This is unlikely, however, because we adjusted the 2003/04 HbA1c values using the calibration equation suggested by NHANES [20] (provided in Methods) and because our results are consistent with those found using self-reported diagnosis of T2DM. Lastly, T2DM is strongly associated with heart disease and heart disease medications, such as diuretics, can affect the clearance of BPA. Thus, because increased BPA levels in urine could be a consequence of treatment of heart disease, the cross-sectional association between BPA and T2DM could be capturing the association between T2DM and heart disease. In our study, subjects who took diuretics had significantly higher concentrations of urinary BPA than those who did not (age, gender, race-ethnicity, and urinary creatinine-adjusted difference in urinary BPA = 1.19 ng/mL (95% CI, 1.08, 1.31), p<0.001). However,inclusion of diuretic use in the model only slightly attenuated the observed associations and did not change the overall patterns of association (data not shown).

Although there is scant evidence of the association between BPA exposure and T2DM or other relevant biomarkers in human populations, the physiological plausibility of BPA's effect on metabolic homeostasis has been explored in both rodent and human tissue culture models and several mechanisms have been proposed. The first is that BPA may lead to overstimulation of the estrogen receptor ERα in pancreatic β-cells. ERα plays a role in increasing insulin biosynthesis in response to increased physiological concentrations of estrogen, such as during pregnancy [5]. Studies in male mice have shown that BPA may also stimulate insulin biosynthesis and secretion through ERα, leading to hyperinsulinemia and insulin resistance [5], [6]. Additionally, genetic defects of ERα are associated with impaired glucose metabolism, insulin resistance, T2DM and metabolic syndrome [29]–[31]. A second proposed mechanism is that BPA mimics estradiol (E2) which is also important to maintaining insulin sensitivity and β-cell function during physiologically relevant periods [32], [33]. Male mice treated with either BPA or E2 show altered glucose tolerance, insulin resistance and hyperinsulinemia [6], [34], [35]. A third mechanism suggested is that BPA may suppress adiponectin, an adipocyte-specific hormone responsible for maintaining insulin sensitivity [36], and that is known to be reduced prior to development of T2DM [37]. Hugo et al. compared the effects of low doses of BPA and E2 on adiponectin secretion from human breast cells, adipose explants and mature adipocytes and found that low doses of BPA and E2 were both effective at suppressing adiponectin release in all tissue types [7]. Similarly, Ben-Jonathan et al. also found that low doses of BPA inhibited adiponectin release in human adipose tissue [8]. This body of evidence suggests that exposure to BPA may lead to altered metabolic homeostasis via multiple pathways.

The strengths of this study include 1) the use of three independent representative samples of the U.S. general population (including oversampled minority populations), which enables the observed results to be generalizable; 2) the use of NHANES data conducted with strict quality control procedures; and 3) the fact that this is the first and largest study of BPA exposure and HbA1c, a stable marker of glycemic index.

Despite HbA1c's benefits, the cross-sectional design used in the NHANES survey may limit causal inferences. Only a single urine sample and a single blood draw were taken for each patient studied in this analysis. While HbA1c is considered relatively stable over several months, BPA is believed to have a short half-life in the human body, with generally all BPA being eliminated within 24 hours of exposure [38]. Exposure assessment using a single urine sample makes it impossible to address the temporal variability of a person's BPA exposure. In fact, BPA concentrations in urine have been found to be highly variable from day to day and the reproducibility has been low [39], [40]. This variability would lead to a non-differential misclassification and therefore bias the association towards the null. Therefore, the use of a single urinary measurement of environmental exposure as a predictor of chronic disease may not be appropriate, especially when the exposure is believed to be relatively short-lived in the human body. While a recent study suggested that the half-life of BPA within the population might be longer than originally believed, the observed stability could also be an effect of continuous widespread exposure in the population, including from non-food sources [41]. Therefore, longitudinal studies, with multiple measures over a much longer time period, will be necessary in order to further investigate if there is a true association between BPA exposure and T2DM.

Additionally, because this study only examined concurrent BPA exposure among adults, it was impossible to investigate any effect that BPA exposure during critical growth periods, such as prenatally, may be having on disease. This is an important research aspect as evidence is mounting that is suggestive that early life exposures may predispose an individual to developing disease later in life [42]–[44]. A recent study in mice even suggested that maternal exposure to BPA could lead to altered metabolic homeostasis in offspring [45]. Male offspring who were exposed to BPA *in utero* were found to have significantly reduced glucose tolerance and increased insulin resistance compared to the offspring of untreated mothers [45]. This would be an important area of future research, given that BPA has been detected in human breast milk, amniotic fluid, placenta, neonatal and cord blood [2], [38].

Given their widespread prevalence in the environment, investigating the role of environmental contaminants like BPA in the pathogenesis of T2DM, obesity and metabolic syndrome is of significant public health importance. While the results of this work have limitations, further study of this possible association is warranted, both to explore possible physiologic pathways involved as well as to understand if long-term exposure and exposure during critical periods of growth and development may play a role in the development of T2DM and other metabolic diseases.
